# Effectiveness and safety of fire-needle moxibustion on insomnia: Protocol for a systematic review and meta-analysis

**DOI:** 10.1097/MD.0000000000014509

**Published:** 2019-02-15

**Authors:** Cuiling Liu, Zhiqiang Chen, Ting Li, Zhihua Yang, Qingsong Zhang, Jianping Yin, Peng Zhou, Wei Fu, BaiShu Chen

**Affiliations:** a Baoan Hospital of Traditional Chinese Medicine in Shenzhen, Shenzhen; b Guangzhou University of Chinese Medicine, Guangzhou, China.

**Keywords:** fire-needle moxibustion, insomnia, protocol, systematic review

## Abstract

**Background:**

Fire-needle moxibustion (FNM) is an ancient method of external therapy that combines acupuncture with moxibustion, and has the property of high temperature resistance. Insomnia is a major public health problem and strongly associated with a high prevalence, impact on daily life, comorbidity with other disorders, and societal costs. The clinical practice demonstrates that FNM has a therapeutic effect on insomnia. Here we will provide a protocol to evaluate the effectiveness and safety of FNM for insomnia.

**Methods:**

We will search the randomized controlled trial literatures of FNM for insomnia in 7 electronic databases, including 3 English databases (PubMed, EMBASE, the Cochrane Central Register of Controlled Trials [Cochrane Library]) and 4 Chinese databases (Chinese National Knowledge Infrastructure, Chinese VIP Information, Wanfang Database, and Chinese Biomedical Literature Database). Pittsburgh Sleep Quality Index will be considered as the primary outcome, and the secondary outcome will include biochemical, indicators total scores on the insomnia severity index, quality of life, adverse events caused by FNM, and changes of TCM syndromes scores. Review Manager 5.2 software will be use for assessment of risk of bias, data synthesis. Begg and Egger tests will be use for assessing symmetries of funnel plot by software Stata 12.0. Methodological quality will be assessed with the risk of bias according to Cochrane Handbook.

**Result:**

This study will provide a rational synthesis of current evidences for Fire-needle moxibustion on insomnia.

**Conclusion:**

The conclusion of this study will provide evidence to judge the effectiveness and safety of Fire-needle moxibustion on insomnia.

**Registration:**

PROS-PERO CRD42019120875.

## 1 Introduction

Insomnia disorder (ID) is the most common sleep disorder in adults with a prevalence range from about 4% to 22%.^[1–3]^ In practice, insomnia is a subjective disorder that manifests as prolonged sleep latency, sleep maintenance disorder, early awakenings, impaired total sleep time, and decline in sleep quality, despite having an adequate opportunity to sleep, associated with distress and daytime dysfunction.^[4,5]^ A cross-sectional telephone survey^[6]^ of 7428 workers in the United States estimated annual insomnia-related work losses of 367 million days and $91.7 billion. Recent studies have found that insomnia is related to higher risk of various cardiovascular disease, cerebrovascular disease, hypertension, and depression.^[7,8]^ In addition, ID is a common command condition in several psychiatric disorders including major depressive disorder, post-traumatic stress disorder, anxiety, alcohol or drug abuse, bipolar disorder, obsessive compulsive disorder, and psychotic disorders.^[9]^

Nowadays, treatments for insomnia are mainly divided into psychotherapy and pharmacotherapy. Cognitive behavioral therapy is recommended as a first-line treatment by the Australasian Sleep Association, which is helpful to improve the quality of sleep.^[10]^ However, few well-trained therapists and poor compliance limited its clinical practice.^[11,12]^ Pharmacotherapy also plays an important part in the treatment of insomnia. Benzodiazepine drugs (BZDs) and nonbenzodiazepine compounds (non-BZDs) are prescribed to treat insomnia. Yet, dependence, abuse potential, withdrawal syndrome, and adverse effects to these drugs for long-term use are also common.^[13]^ Hence, there are number of insomniacs seeking remarkably curative with less adverse effects for insomnia.^[14]^ As an alternative or complementary treatment, acupuncture and moxibustion are considered helpful in improving symptoms of insomnia.

Fire-needle moxibustion (FNM) is a combination therapy of acupuncture and moxibustion, which has been widely used in China.^[15–17]^ The tip of the fire needle is round that could increase the contact area with the lesions. What's more, the fire needle has the property of high-temperature resistance. The needling depth, acupuncture manipulation, and needle retention time are different from traditional acupuncture such as filiform needle and warm needle. Skilled Traditional Chinese Medicine (TCM) practitioners use the needle to prick the selected acupoints by depth of 0.3 to 0.5 cm and then remove the needle swiftly. FNM has a synergistic effect of heat from moxibustion and stimulation on acupoints in promoting calcification, improving blood circulation, and eliminating blood stasis,^[18,19]^ especially within a narrow range around the fire-needle body, the lesions were burnt to carbonization.^[20]^ Some clinical trials have found that FNM has a significant impact on insomnia. However, the randomized controlled trials (RCTs), reviewing the efficacy and safety of FNM for insomnia, have not been systematically summarized. Thus, the purpose of this review is to summarize clinical researches on FNM for insomnia, and findings of this review will be reliable within evidence of clinical studies. This review only focuses on the effects of FNM on insomnia rather than other effective treatments.

## 2 Methods

### 2.1 Study registration

The protocol has been registered on the International Prospective Register of Systematic Reviews (PROSPERO) (registration number CRD42019120875) basing on the Preferred Reporting Items for Systematic Reviews and Meta-Analyses Protocols (PRISMA-P) statement guidelines^[21]^ on January 17, 2019.

### 2.2 Inclusion criteria and exclusion criteria

#### 2.2.1 Research type

We will merely include the RCTs which evaluated the efficacy of FNM in treating insomnia, regardless of blinding, publication status, or language. International criteria for diagnosing insomnia will also be included^[22]^: complaints of difficulty in falling asleep or early awakening; refractory sleeping difficulties, in spite of adequate sleeping opportunities and proper environment; daytime dysfunctions caused by dyssomnia.

#### 2.2.2 Participant type

Patients who have been diagnosed with insomnia according to the diagnostic criteria and relevant test results, irrespective of sex, age, severity, and time of illness, will be selected as the research objects.

#### 2.2.3 Intervention measures

Experimental group will include FNM as intervention measure alone or FNM combined with normal acupuncture, decoction, or Western medicine as intervention therapy. Control group will include common acupuncture as intervention measures, and sedative-hypnotic medicine for insomnia will be taken into consideration.

#### 2.2.4 Major research indicators

The evaluation indicators are as follows: primary outcomes will reflect in the average difference in Pittsburgh Sleep Quality Index (PSQI) after treatment. Secondary outcomes will include total effective rate, TCM syndrome effect, and adverse events like dizziness, nausea, and vomiting.

#### 2.2.5 Exclusion criteria

Studies with the following features will be excluded: non-RCT literatures; patients whose baseline data are significantly inconsistent; studies whose treatment measures fail to meet the requirement of warming-needle moxibustion; studies involving noninsomnia patients; incomplete case reports, animal experiments, reviews, conference papers or important data reports with no reply from the corresponding author(s).

### 2.3 Search methods for study identification

#### 2.3.1 Electronic searches

**Figure 1:**
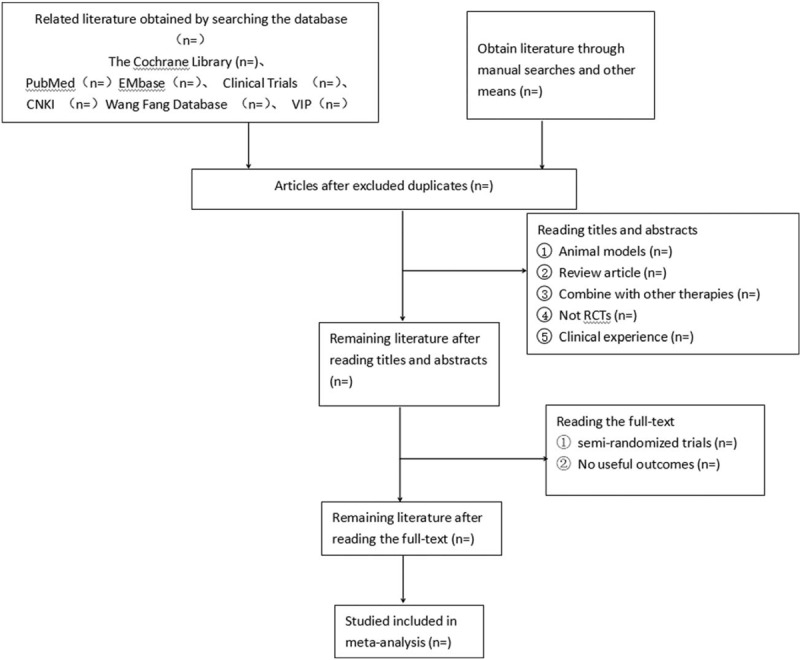
Flow chart of the study selection procedure.

According to the Preferred Reporting Items for Systematic Reviews and Meta-analyses (PRISMA), 7 clinical studies databases, including PubMed, Embase, The Cochrane Library, China National Knowledge Infrastructure, VIP, and Wanfang database will be searched to identify RCTs (Fig. 1). The time span will start from the establishment of each database and end in January, 2019. The keywords being used are as follows: Chinese keywords will be Chinese pinyin such as “Huozhen” (which means “Fire-needle moxibustion ”) and “Shimian, Bumei, Bumian and Budewo” (which means“insomnia”); while English searches combined subject terms (MeSH) and free words, with a retrieval strategy of “insomnia”or“sleeplessness”or“DIMS (Disorders of Initiating and Maintaining Sleep)”AND“Fire-needle moxibustion.”

#### 2.3.2 Searching other resources

Relevant systematic review or meta-analysis of RCTs on FNM will be electronically searched. Moreover, we will filter relevant medical journals and magazines to identify literature which is not included in the electronic databases.

### 2.4 Data selection and extraction of relevant data

After researching the selected literature carefully, 2 reviewers (Chen ZQ and Li T) will come to retrieve documents from the databases mentioned above. Documents possibly relevant to the study will be selected after a preliminary analysis of abstracts and primary coverage of these documents. Then, a new round of selection will be conducted via reading the full text of each document. Finally, the papers will be cross-checked by the 2 investigators and reach a consensus by discussions or opinions from a third party. Lacked information will be supplemented by communicating with authors of these studies. Data extraction will include: the first author, time of publishing, random method, sample size of intervention measures and controlled measures, outcomes, course of treatment, and assessment criteria.

### 2.5 Data analysis

#### 2.5.1 Risk assessment of included studies

Cochrane Handbook, version 5.1.0 will be utilized to assess selected RCTs from the perspectives of stochastic series generation, allocation concealment, implementation of the blind method, incomplete data, selective report, and other bias. The evaluation criteria of each item will be judged as “low risk of bias,” “unclear risk of bias,” and “high risk of bias.”

#### 2.5.2 Statistical analysis

Statistical software (Review Manage software [RevMan 5.3] and Stata12.0) will be implemented to perform this meta-analysis. For continuous variables, mean difference (MD) will be used to evaluate the extracted data. For dichotomous variables, rate ratio will be applied to analyze. 95% confidence intervals (CIs) for both continuous and dichotomous variables will be set up.

#### 2.5.3 Dealing with missing data

For insufficient or missing trial data, we will try to contact the corresponding author through multiple avenues. On condition that the missing data cannot be supplied or failure to contact the author, a limited analysis based on the available data and discuss the potential impact of the missing data will be perform.

#### 2.5.4 Assessment of heterogeneity

We are evaluating the heterogeneity with *I*^*2*^ values according to the Cochrane Handbook (0%–40%, less important; 30%–60%, representing moderate heterogeneity; 50%–90%, representing substantial heterogeneity; and 75%–100%, representing considerable heterogeneity). If the heterogeneity among trials is significant (*I*^2^ ≥ 50%), the random-effects model will be selected and further subgroup analysis will be performed to investigate the possible causes of heterogeneity. On the contrary, if the *I*^2^ value is less than 50%, we will choose the fixed-effect model.

#### 2.5.5 Assessment of reporting bias

The presence of reporting bias will be evaluated by funnel plots when studies included in the review are sufficient (more than 10 trials). It will be considered that the reporting bias is existing and the reliability is low if the points on both sides of the funnel plot are dispersed and asymmetrical. Conversely, if the points on either side of the funnel plot are symmetrically distributed in substantial, the reporting bias will be considered as nonexistent and the result is reliable.

#### 2.5.6 Data synthesis

All analyses will be conducted by using RevMan software (V5.3, The Nordic Cochrane Centre, The Cochrane Collaboration, Copenhagen, Denmark). We will select a random-effects model or fixed-effects model to merge the primary and secondary outcome indicators in accordance with the results of heterogeneity test. The fixed-effects model will be applied for data synthesis of low heterogeneity (*I*^2^ < 50%), whereas the random-effects model will be conducted if the heterogeneity is significant (*I*^2^ ≥ 50%). It is considered that differences are statistically significant if the results of *Z* test show that *P* value is less than .05, and the 95% CI does not contain 0 (for continuous variables) or 1 (for dichotomous variables).

#### 2.5.7 Subgroup analysis

If heterogeneity is evaluated as significant (*I*^2^ ≥ 50%) and the trials included are adequate, we will perform a subgroup analysis to explore the potential source of the heterogeneity according to the difference in participant characteristics, interventions, controls, and outcome measures.

#### 2.5.8 Sensitivity analysis

According to sample size, methodological quality, and the effect of missing data, sensitivity analysis will be carried out to identify the quality and robustness of the meta-analysis result when the outcome analyses involve a large degree of heterogeneity.

#### 2.5.9 Grading the quality of evidence

We will evaluate the quality of evidence and rate it into four levels: high, moderate, low, or very low according to the Recommendations Assessment, Development and Evaluation guideline.^[23]^

#### 2.5.10 Ethics and dissemination

Ethical approval will not be necessary because the data being included in our study are derived from published literature and will not be linked to individual patient data. The systematic review providing implication of the effectiveness and safety of FNA for insomnia will be published in a peer-reviewed journal or conference presentations.

## 3 Discussion

Insomnia is the most prevalent sleep disorder and shows an independent risk factor for several diseases, such as diabetes mellitus, cardiovascular disease, affective disorder, and so on.^[24]^ The pharmacotherapies for insomnia can cause adverse effects, whereas placebo effects of the pharmacological therapy should not be neglected, and the ideal medication is undiscovered at present. Therefore, it is necessary to search for alternative therapy with higher effective rate and less side effects.

First-line drugs including benzodiazepines and benzodiazepine receptor agonists are widely applied nowadays. However, observational studies have shown that hypnotic drugs may be associated with infrequent, but serious, adverse effects such as dementia, serious injury, and fractures.^[25–28]^ FNM is the combination of acupuncture with moxibustion, which has been applied in numerous clinical conditions, including insomnia and psychiatric disorders.^[29–33]^

To our knowledge, this will be the first meta-analysis focusing on efficacy and safety of FNM on insomnia compared with control group. This meta-analysis will examine the evidence for the safety and efficacy of FNM for the management of insomnia. Our analysis will show patients’ changes in PSQIs, clinical efficacy, and TCM syndromes after receiving FNM therapy. Adverse reactions and side effects caused by FNM will also be shown. Thus, the results will offer reliable references for clinicians and patients in the treatment of insomnia with FNA. Last, but not least, the results may introduce an alternative therapy of insomnia to policy makers to decrease the burden of public health.

## Author contributions

BaiShu Chen conceived the study idea. CuiLing Liu were responsible for the design of this systematic review. ZhiQiang Chen, Ting Li and Peng Zhou contributed to the data analysis plan. ZhiHua Yang and QingSong Zhang drafted the manuscript and JianPing Yin, Wei Fu edited. All authors provided feedback and approved the final manuscript.

**Conceptualization:** Cuiling Liu, BaiShu Chen.

**Data curation:** Zhiqiang Chen.

**Formal analysis:** Ting Li.

**Investigation:** Zhihua Yang.

**Methodology:** Qingsong Zhang.

**Project administration:** Jianping Yin.

**Software:** Peng Zhou.

**Writing – original draft:** Cuiling Liu.

**Writing – review & editing:** BaiShu Chen, Wei Fu.
